# Research ethics committees: agents of research policy?

**DOI:** 10.1186/1478-4505-3-6

**Published:** 2005-10-04

**Authors:** Elina Hemminki

**Affiliations:** 1 Health and Social Services, National Research and Development Centre for Welfare and Health STAKES, P.O. BOX 220, 00531 Helsinki, Finland

**Keywords:** ethics committees, clinical trials, ethics, law

## Abstract

The purpose of this commentary is to describe the unintended effects ethics committees may have on research and to analyse the regulatory and administrative problems of clinical trials.

**Discussion:**

The Finnish law makes an arbitrary distinction between medical research and other health research, and the European Union's directive for good clinical trials further differentiates drug trials. The starting point of current rules is that clinical trials are lesser in the interest of patients and society than routine health care. However, commercial interests are not considered unethical. The contrasting procedures in research and normal health care may tempt physicians to continue introducing innovations into practice by relying on unsystematic and uncontrolled observations. Tedious and bureaucratic rules may lead to the disappearance of trials initiated by researchers. Trying to accommodate the special legislative requirements for new drug trials into more complex interventions may result in poor designs with unreliable results and increased costs. Meanwhile, current legal requirements may undermine the morale of ethics committee members.

**Conclusion:**

The aims and the quality of the work of ethics committees should be evaluated, and a reformulation of the EU directive on good clinical trials is needed. Ethical judgement should consider the specific circumstance of each trial, and ethics committees should not foster poor research for legal reasons.

## Introduction

Various ethical rules and, lately, an increasing amount of legislation have been introduced to protect research participants. Ethical codes target the researchers, but ethical committees have come into existence to aid the researcher and to ensure that rules are followed. Ethical rules and codes have emerged within medical research, and still today in most countries, the legal requirements for research protocols to be checked by ethics committees are confined to medical research.

Clinical trials are used variously to obtain unbiased estimates of the value of health technologies, for administrative purposes (e.g. for registering medicines), to get opinion leaders to commit, and for marketing. Currently, most clinical trials concern new drugs or new indications for drugs. Dorman et al.'s [1] analysis of trials in acute stroke from 1955–95 showed that only 12% tested non-drug interventions. Active and successful research in biomedicine along with powerful financing – most commonly by the drug firms – have made drug trials a central element of clinical research.

The main aim of ethics committees is to protect patients involved in research. However, it has been asked whether the committees do more harm than good, with researchers expressing concern at the negative impact on research [see for e.g. [2,3]]. Experts have identified serious problems in the processes [4,5,3], while a great variability in the criteria used by the committees is reported both within and between countries [6-10,3], with most of the empirical studies looking at ethics committees come from the U.K. The problems listed include too much work for voluntary committee members, slowness (crucial for studies with time-limited budgets), varying criteria used by different committees, a lack of training of the members of ethics committees, repeat handling of multicenter trials, and excessive costs. Less criticism has been made of omissions – what is allowed to happen that should not be [11], or the waste of limited clinical research capacity on trivial research [12].

The purpose of this article is to describe the unintended effects ethics committees may have on research, using Finland as an example and focusing on clinical trials. I will analyse some of the regulatory and administrative problems associated with clinical trials and suggest solutions.

Finland is a Northern European welfare state with a population of about 5 million people. Trials of new drugs are common in Finland (tenfold higher than might be expected on the basis of the size of the Finnish population) [13], with 287 new drug trials reported to the drug regulatory authority in 2001 [14].

This article is based on observations drawn from reports of clinical trials, both published and unpublished; authoritative advice for conducting trials; my own experience of submitting research to ethics committees, judging research protocols in funding organizations and sitting in Finnish ethics committees. Much of my experience is based on material that is, or has been, confidential, and so I will amalgamate the information for purposes of anonymity.

## Discussion

### The regulation of clinical trials and ethics committees

In Finland, ethics committees were first established voluntarily in the 1970s, whilst later their tasks and composition were regulated through a 1999 Act on medical research [15,13]. Only medical research ("research, which affects human integrity and is led by physicians") is expected to be submitted to ethics committees for prior evaluation. The European Union good clinical trials directive [16] was integrated into the Finnish national law in spring 2004, differentiating drug trials as a subgroup that is subject to more detailed regulation. In addition, the directive added many administrative and surveillance tasks in regard to drug trials, some of which are only vaguely related to ethics, such as insurance, compensation of injuries, and external surveillance of adverse drug events.

The Finnish law requires that informed consent is obligatory in all drug trials, and the procedures are regulated in detail; in non-drug clinical trials, informed consent may be waived in exceptional circumstances. Informed consent is defined such that the people who are "the targets" of the trial will be included in the study only after the trial has been described to them honestly and in detail, and they have then voluntarily given their permission, free of any pressures. The consent "must be written, dated and signed", and in the case of incapable persons, this should be made by his or her legal representative. If the person cannot write, oral consent may be accepted in the presence of at least one witness [16]. The Finnish Act (Sublaw 986/1999) gives a detailed list of the formal requirements of informed consent and additional details on how to ask for informed consent and which kinds of documents are needed are given by drug control authorities and the central ethics committee. One requirement is that a copy of the signed consent is to be given to the patient or other research participant, suggesting a contract arrangement.

Informed consent on data acquisition and its use is much vaguer. In general, the legal and ethical issues of data use in health research are a muddle due to law changes over time, historic data sets, commercial interest, and special questions of genetic data or other biological samples. In multinational studies, the issue is complicated by the effects of varying data protection laws and their interpretation in different countries. Many researchers have decided to avoid the problems by asking for informed consent from the patient to also use his/her data outside the current research frame. This consent is likely to be uninformed, because an average patient does not know, for example, what information can be obtained from a blood sample or what data national registers contain, or what does anonymity of genetic samples imply. In clinical trials, informed consent for data storage or transfer often ends up being a quasi-action from an ethical point of view.

Current ethical rules in Finland do not classify commercial interests as unethical, and they do not need to be revealed to trial participants. Patients may be asked to join trials with designs favouring the studied therapy or they are asked to join trials intended to accustom physicians to prescribing a certain therapy through their trial participation. A large number of trials remain unpublished (within pharmaceuticals, this is more than half), and those favouring the therapy being studied are more likely to be published [17-22]. A common motive for patients to take part in research is an altruistic wish to help medicine develop for the common good, and were the patients to know that the trial was created for commercial reasons and/or the results would remain unavailable, they may not have participated.

### Double standards on informed consent

Current rules for Finnish ethics committees – for example, as expressed in the EU directive on good clinical trials – start from the idea that clinical trials are, by definition, lesser in the interests of the patient and society than routine health care. The ethical codes do not explain why an intervention that is already used in patient care automatically requires an informed consent within a research setting [23-25]. In ordinary health care, the same intervention may be prescribed by less experienced and knowledgeable practitioners. In the words of the paediatrician Richard Smithells: "I need permission to give a drug to half of my patients, but not to give it to them all" [cited by [23]].

Even though the idea of a physician being a consultant to help the patient make an informed choice has been put forward, it is likely that this consumerism in health care will only expand into issues where lay-persons have the main responsibility for health decisions, such as in the field of prevention and some chronic diseases. In medical research, current ethics have adopted the consumer model, largely putting the responsibility on the patient regardless of the nature of the issue being studied.

The contrast between the procedures required in research and normal health care is striking. Additional research requirements may tempt physicians to continue the old method of introducing innovations into practice by relying on unsystematic and uncontrolled observations. In the case of new drugs, the inflexible requirement of informed consent in emergency situations with critically ill patients may be unethical and/or unfeasible. It may result in the future with emergency medication being based on uncontrolled experimentation.

In practice, asking for informed consent is often something of a performance, in which patients and physicians are acting as if a truly informed consent was asked for. In many situations, patients cannot or prefer not to make an informed choice [26]. Moreover, even if such an attempt at truly informed consent is made, it is not often successful. Diseases and ill health are concentrated among the aged. Most patient information leaflets and consent forms are long with difficult language [27,28].

### Impact on ethics committees

In many countries, ethics committees have been transformed from bodies providing advice into administrative bodies observing that rules are followed [for the U.K., see [29]]. If in conflict, the law precedes ethics. The legal requirements used in judging the ethicality of clinical trials may undermine the morale of the members of ethics committees. Giving positive statements on research protocols fulfilling legal requirements, yet wasting resources and bringing no value to health may create cynicism and decrease true interest in their work. Such situations include accepting marketing research disguised as scientific research, or accepting patient information leaflets which contain all the necessary information, but which are unlikely to be understood by the target group. Giving a negative statement on important ethical research that does not fulfil legal requirements may also undermine morale.

### Impact on research orientation

Tedious and bureaucratic rules may result in more and more incentives being necessary to persuade physicians and their employers to carry out trials, making trials more expensive. Trials initiated by researchers may disappear and only those trials having a rich sponsor will survive. We are already in a situation where most drug trials are paid for by drug companies [1,30-33]. In the U.K., non-commercial sources have also extensively supported clinical trials, including non-drug trials, but in recent years there has been a clear decline [34].

"Ethical" codes and legislation may lead to trials becoming tedious, expensive and factory-like, alienating interested minds and health service providers. In the worst scenario, research resources are wasted, answers are received to unimportant questions, and scientists turn to other types of health research.

The current Finnish legislation and many international codes have been made to accommodate the special requirements of new drug trials and they do not fit well into established therapies or into more complex interventions, such as prevention and ways of organizing services. Trying to accommodate the legal requirements may result in poor designs with unreliable results and increased costs, and interest in studying complex interventions may diminish. For example, if the requirement of informed consent is interpreted rigidly, cluster randomization would become unfeasible.

## Conclusion

Comparative trials that answer important health or health service questions and which are not biased by commercial interests are needed. They should be applicable to real life situations, and use resources prudently to allow many questions to be studied.

The research question and field circumstances should determine how clinical trials should be done. When the Helsinki Declaration was formulated, Bradford Hill [35], an innovator behind clinical trials, claimed that there is no one way of doing clinical trials ethically, and giving detailed advice as if there were will harm both research and ethics. He argued that ethical judgement has to consider the specific circumstances of each trial. General advice for trial design and ethics are useful in giving inexperienced researchers help in their work, but as Foster [29] has argued, to decide what is ethically appropriate requires a thoughtful balancing between different moral approaches and cannot simply be substituted by regulation and rules.

It seems that ethics committees have sometimes become an extra burden instead of an aid to bettering clinical trials. The external review by ethics committees should be advisory and they should not be censoring and preventing research, but advising and helping researchers to carry out responsible research. Ethics committees should judge the ethical components, free from rigid detailed rules, guided by general principles, enriched in international debate. Ethics include the fair use of health care resources and the potential value of the study. The burden of judging the benefits and risks should not be put solely onto individual patients via informed consent.

The concept and practical application of informed consent should be rethought for trials with interventions which can be used without informed consent in everyday practice. Normal health care and research on existing practices should have similar ethical rules [24,25]. Opting out and "non-compliance" are the rights of a person, both in research and normal care. But people cannot (individually) beforehand decline from being asked to enter mass screening, or choose hospital wards randomized to varying (established) treatment policies. Their role in deciding on emergency care is limited, too. Whether or how individuals are informed about the trial should depend on the intervention. For screening, Irwig et al. [36] have proposed a survey of the target population's interest in participating after being fully informed before the offer of screening. For trials comparing different treatment policies (pragmatic trials), providing information to and gaining permission from the communities in which the research is being carried out is an option. For collective permission to be useful, it may, however, need public education on what the trials are and why they are valuable.

Participation in trials with non-commercial interests should be seen as a professional responsibility [37], and clinical trials with existing therapies or service provision should be considered a part of health services.

The role of ethics committees should be expanded to cover commercial interests. Ethics committees should guarantee that all potential participants, both physicians and patients, are aware of the financer, and what, if any, are the commercial aims, as well as what compensation is paid, and whether results will be publicly available.

The rules and legislation governing the work of ethics committees as well as the quality of their work should be evaluated. Observations suggest that ethics committees are doing tasks which do not suit them, and which prevent them from concentrating on the real issues. Furthermore, there is a worry that the new EU legislation may worsen the opportunities to do trials which are in the patients' interest. An urgent task in Europe is to reformulate the EU directive on good clinical trials and to discuss ethics from a wider perspective.

## Competing interests

The author(s) declare that they have no competing interests.
